# Antibiotic De-Escalation in Emergency General Surgery

**DOI:** 10.3390/antibiotics11091148

**Published:** 2022-08-24

**Authors:** Carlo Vallicelli, Margherita Minghetti, Massimo Sartelli, Federico Coccolini, Luca Ansaloni, Vanni Agnoletti, Francesca Bravi, Fausto Catena

**Affiliations:** 1 General, Emergency and Trauma Surgery Department, Bufalini Hospital, 47521 Cesena, Italy; 2 Department of Surgery, Macerata Hospital, 62100 Macerata, Italy; 3 General, Emergency and Trauma Surgery Department, Pisa University Hospital, 56124 Pisa, Italy; 4 Department of General and Emergency Surgery, Policlinico San Matteo, 27100 Pavia, Italy; 5 Anesthesia, Intensive Care and Trauma Department, Bufalini Hospital, 47521 Cesena, Italy; 6 Healthcare Administration, Santa Maria delle Croci Hospital, 48121 Ravenna, Italy

**Keywords:** antibiotics, de-escalation, emergency general surgery

## Abstract

*Background.* Antibiotic treatment in emergency general surgery (EGS) is a major challenge for surgeons, and a multidisciplinary approach is necessary in order to improve outcomes. Intra-abdominal infections are at high risk of increased morbidity and mortality, and prolonged hospitalization. An increase in multi-drug resistance bacterial infections and a tendency to an antibiotic overuse has been described in surgical settings. In this clinical scenario, antibiotic de-escalation (ADE) is emerging as a strategy to improve the management of antibiotic therapy. The objective of this article is to summarize the available evidence, current strategies and unsolved problems for the optimization of ADE in EGS. *Methods.* A literature search was performed on PubMed and Cochrane using “de-escalation”, “antibiotic therapy” and “antibiotic treatment” as research terms. *Results.* There is no universally accepted definition for ADE. Current evidence shows that ADE is a feasible strategy in the EGS setting, with the ability to optimize antibiotic use, to reduce hospitalization and health care costs, without compromising clinical outcome. Many studies focus on Intensive Care Unit patients, and a call for further studies is required in the EGS community. Current guidelines already recommend ADE when surgery for uncomplicated appendicitis and cholecystitis reaches a complete source control. *Conclusions.* ADE in an effective and feasible strategy in EGS patients, in order to optimize antibiotic management without compromising clinical outcomes. A collaborative effort between surgeons, intensivists and infectious disease specialists is mandatory. There is a strong need for further studies selectively focusing in the EGS ward setting.

## 1. Background

Antibiotic resistance is a major global issue, and the appropriate use of antibiotics is a cutting-edge topic in the current literature. Abdominal acute care and emergency surgery is a particularly challenging situation for the proper management of antibiotic treatment. Intra-abdominal infections (IAI) are known to be associated with high morbidity and mortality, prolonged hospitalization and increased hospital costs. Surgeons often face complex clinical scenarios in emergency general surgery (EGS). Patients presenting with IAI have an increased fragility due to the infectious condition itself, that may be further aggravated by the presence of comorbidities or immunosuppression. Moreover, patients with IAI may be at an increased risk of multi-drug resistant bacterial infections in case of recent hospitalization or recent surgery. When facing an emergency surgery patient, antibiotic treatment should be balanced together with source control strategies, in a relationship often challenging to manage and with timing that is often difficult to define. Of course, antibiotics are a cornerstone in the treatment of emergency general surgery (EGS) conditions. However, in this complex setting, there is an increased risk of antibiotic misuse and over-use. A rate of up to 47% of inappropriate antibiotic prescriptions has been documented in surgical specialties [1,2,3]. This may lead to antibiotic resistance and to an increased rate of infections caused by multi-drug resistant bacteria, which are responsible for increased morbidity, prolonged hospitalizations and higher health-care costs. Appropriate antibiotic treatment is therefore of utmost importance in EGS and the role of antibiotic de-escalation (ADE) should be strongly highlighted in this setting. Once an empiric antibiotic treatment is established, frequent re-assessments in the context of ward rounds and antibiotic stewardship programs should lead to an optimization of the treatment as soon as possible. Surgeons are known to be reluctant to accept recommendations on antibiotic therapy; in particular, they have lower odds of agreement concerning de-escalation while they prefer recommendations concerning improvement in the antimicrobial spectrum [4]. This evidence suggests the need for a revolution in the way of thinking within the surgical community. Therefore, the topic of ADE should be increasingly studied and evaluated, especially in the EGS setting.

## 2. Methods

A literature search was performed on PubMed and Cochrane to identify suitable publications using the following search terms: “de-escalation” AND (“antibiotic therapy” OR “antibiotic treatment”). The searches were limited to papers fully published in English. The resulting outputs were combined, excluding duplicate results. Abstracts were scanned for suitability and the full text retrieved for all potentially relevant studies. Bibliographies and reference lists were reviewed to identify additional relevant studies.

The inclusion criteria for the studies were as follows: (1) ADE of antimicrobial therapy, (2) application of any intervention or provision of substantial epidemiological data to judge the effects or determinants of ADE.

## 3. Antibiotic De-Escalation: Meaning and Definition

The first issue when talking about ADE is the absence of a globally accepted consensus definition. Although commonly defined as a narrowing of the spectrum of antimicrobial treatment, ADE has also been identified in other forms. ADE has been described as a decrease in the number of antibiotics used in a treatment regimen or as a shortening in the duration of the therapy [5]. Moreover, ADE has also been defined as stopping combination therapy [6] or switching antibiotics from an intravenous to oral route [7] (Table 1).

## 4. Current Evidence on Antibiotic De-Escalation in Emergency General Surgery

According to international guidelines, the use of broad spectrum antibiotics is the cornerstone of the empirical treatment of critically ill EGS patients, in order to minimize the risk for inadequate antimicrobial therapy [8,9,10]. However, this strategy has the drawback of increased antibiotics-related side effects, extra costs, and bacterial resistance. Several studies recommend ADE as soon as microbiological information is available. Garnacho-Montero et al. conducted a prospective observational study on patients admitted to Intensive Care Units (ICU) with sepsis or septic shock. A total of 219 patients were enrolled and 35% of them received ADE. The hospital mortality rate was significantly lower in ADE strategy, as compared to no treatment change or treatment escalation. Multivariate analysis revealed the ADE strategy to be protective for hospital 90-day mortality in the entire cohort [7]. Turza et al. retrospectively reviewed 2658 admitted to surgical ICU with lung, abdominal and urinary tract infections. ADE was not associated with an increased mortality as compared to no-ADE strategy [11]. The Short Course Antimicrobial Therapy for Intra-abdominal Infection (STOP-IT) trial evaluated 518 patients with complicated IAI undergoing adequate source control. Similar outcomes were demonstrated after a fixed-duration of antibiotic therapy (approximately 4 days) as well as after a longer course of antibiotics (approximately 8 days) that was extended until after the resolution of physiological abnormalities [12]. In the DURAPOP trial, 249 patients with postoperative IAI were randomized into two group. In the first group, antibiotic therapy was stopped at day 8, while in the second one was continued until day 15. There was no significant difference in clinical outcomes [13]. Montravers et al. selectively reviewed a population of consecutive health-care associated IAI patients admitted to ICU, showing no differences in Sequential Organ Failure Assessment (SOFA) score changes and mortality between ADE and no-ADE groups. Postoperative peritonitis was defined as the first macroscopic finding of IAI combined with positive fluid culture at the time of reoperation after a first abdominal surgery. The authors enrolled 206 patients and ADE was performed in 110 of them (53%). No clinical difference was observed at day 7 between the two groups. Determinants of ADE at multivariate analysis were adequate empiric therapy and empiric use of vancomycin, carbapenems and aminoglycosides. Cultures growing non-lactose-fermenting gram-negative bacilli and multi-drug resistant bacteria were less likely to be de-escalated. The authors conclude that ADE is a feasible option in patients with polymicrobial IAI [14]. Koupetori et al. enrolled patients from 2006 to 2013 and divided them up in two periods (2006–2009 and 2010–2013), then compared the ADE and non-ADE groups for both periods. Kaplan–Meier analysis showed no statistical difference in final outcome for the first period, but, concerning the second period group, prolonged survival was found for the de-escalated subgroup [15]. In a 2016 literature review, Tabah et al. compared mortality rates between ADE and non-ADE groups in 1688 patients. The pooled estimated mortality showed a protective effect of ADE (RR 0.68). None of the studies reviewed reported a worse survival in the ADE group. Isolation of multi-resistant pathogens, polymicrobial infections and IAI was a factor negatively associated with ADE [5]. A retrospective analysis of 929 patients with intra-abdominal infection showed that antibiotic therapy shorter that 7 days was associated with the same mortality rate and lower recurrence rate than longer therapies [16]. Moreover, guidelines investigate the proper duration of antibiotic therapies. Infectious Disease Society of America (IDSA) guidelines, concerning patients with complicated intra-abdominal infections, suggest to limit therapy to 4–7 days. In addition, these guidelines focus on some conditions, among which, only a prophylaxis is necessary instead of a treatment of infection such as traumatic or iatrogenic bowel injuries operated on within 12 h, upper gastrointestinal perforations operated on within 24 h and localized process (non-perforated appendicitis, cholecystitis, bowel obstruction and bowel infarction) [17]. Based on the above mentioned definition, studies available on ADE mostly focus on ICU patients. It has been estimated that the average volume of antibiotic consumption in this population is 1563 defined daily doses per 1000 patient-day, almost three times higher than in ward patients [18]. Indeed, EGS patients often require a postoperative ICU stay, especially in the most complex surgical situations. These are patients at an increased risk not only for IAI or intra-abdominal complications, but also for medical complications such as pneumonia, urinary tract infections and bloodstream infections. Therefore, when choosing the proper antibiotic regimen, a multi-disciplinary vision is necessary, and as a consequence, ADE should be tailored to every single EGS patient after a comprehensive evaluation. It is clear that further studies are required, and particularly a call for studies selectively focused on EGS patients not necessarily admitted to ICU is warranted in the acute care and emergency surgery community (Table 2).

## 5. De-Escalation Strategies in Emergency General Surgery

De-escalation strategies may differ in many aspects. Implementing an empirical antibiotic coverage is pivotal to cure patients with sepsis, but it has to be targeted as soon as possible. So, it is mandatory to obtain appropriate cultures before starting an antimicrobial therapy in order to identify the pathogens. In addition, broad spectrum therapy should treat the entire possible microorganism responsible for septic status. Obviously, once the pathogens are identified, the therapy has to be narrowed. De-escalation strategies include shortening the duration of the therapy and source control, as well as narrowing antimicrobial therapy on the results obtained through the cultures. Surgeons must maintain a high warning level when managing an EGS patient. Multiple cultures should be obtained intra and postoperatively. Ideally, antimicrobial therapy should be directed at the common microbiota of the suspected source of sepsis. For IAI, factors known to be associated with worse outcomes and nosocomial or multi-drug resistant pathogens are length of hospital stay or recent health-care exposure, recent antibiotic treatment and recent abdominal surgery [19]. When resistant Gram-positive bacteria (such as MRSA or Enetrococcus faecium) are not isolated in microbiological samples, antimicrobial therapy against them should be stopped. Whenever a Gram-positive bacteria susceptible to beta-lactams antibiotics is isolated, therapy should be switched towards an agent with a narrower spectrum [7]. Taking into account Gram-negative bacteria, several studies demonstrated the effectiveness of monotherapy with no negative clinical impact [7,20,21]. Therefore, if two active antimicrobials were empirically initiated to cover Gram-negative bacilli, current evidence suggests that treatment should be safely switched to monotherapy. The only exception to this recommendation is represented by carbapenemase-producing Klebsiella–Pneumoniae [22]. Moreover, whenever possible carbapenems should be stopped and switched to another antimicrobial with a narrower spectrum, and should be strictly and accurately reserved as they often represent the only therapeutic option towards beta-lactamase producing Enterobacteriaceae, Pseudomonas aeruginosa and Acinetobacter baumanii. Antifungal therapy is empirically started especially in EGS patients with a perforation of the upper gastrointestinal tract. Antifungal therapy should be stopped if fungi are not present in microbiological samples. Echinocandins are the treatment of choice in critically ill patients with invasive candidiasis. Therapy should be continued in patients with candidemia until 14 days after the first negative blood culture. If the patient is clinically stable and the culture has become negative, it is recommended to de-escalate to oral fluconazole after 10 days of intravenous treatment [23]. A formal infectious disease consult is always recommended in case of candidemia.

Cultures results need usually at least 48 h to be ready. They can be deemed unreliable in case of previous antibiotic exposure. Moreover, a 30–80% rate of negative cultures in patients clinically considered infected has been reported. Molecular diagnostic solutions have been proposed to speed the process up such as PCR array merged for specific clinical contests (i.e., pneumonia, bloodstream infections). Two disadvantages of these technologies are costs and the need for blood cultures to establish antibiotic susceptibility, since only a limited number of acquired resistance genes can be screened.

## 6. Pharmacokinetics and Pharmacodynamics

Antibiotics stewardship, ward rounds and frequent antibiotic time-outs are a fundamental part of ADE strategies. Evaluation of pharmacokinetics and pharmacodynamics are of utmost importance since they can fluctuate significantly in critically ill EGS patients. Optimization of dosing strategies include the optimization of the time the serum level of beta-lactams antibiotics are above the mean inhibitory concentration (MIC), as well as optimizing the peak serum level above the MIC for fluoroquinolones and aminoglycosides. It has been estimated that one out of six patients receiving beta-lactams does not reach the minimal concentration target and many more do not reach the target associated with maximal bacterial killing [24]. Utilization of less commonly used antibiotics and alternating dosing strategies can also help in preventing antibiotics resistance of bacteria. Moreover, therapeutic drug monitoring by measuring drug concentration is part of ADE strategies. It is particularly indicated for aminoglycosides and vancomycin due to their high individual pharmacokinetic variability [25].

## 7. Source Control

Another fundamental part of ADE strategies in EGS is proper source control. Source control is defined as elimination of infective foci through drainage, debridement, device removal, compartmental syndrome decompression and deferred definitive restoration of anatomy and function. Source control can be both surgical and interventional in abdominal emergencies. The appropriateness of the source control does not only rely on the completeness of the abdominal infection control but also on a timely strategy. Source control should be obtained as soon as possible, especially in critically ill and septic patients. In-hospital delay of source control has demonstrated a worsening of the outcome in several clinical scenarios. The efficacy of source control is time-dependent and is a major determinant of outcome regardless of the effectiveness of antimicrobial therapy [25].

## 8. Biomarkers

Biomarkers can help in identifying bacterial infections and guiding the treatment strategy. Procalcitonin (PCT) levels are high in bacterial infections, while they remain low in viral infections or non-infectious systemic inflammatory syndromes. PCT could be a useful aid in choosing the proper duration of antibiotic therapy [26]. Prolonged duration of antibiotic therapy has been associated with the emergence of antimicrobial resistance [27]. Eleven randomized trials on PCT-guided antimicrobial treatment have shown a significant reduction in antimicrobial consumption if treatment was guided by PCT levels [16]. When PCT level decrease to normality, antibiotic treatment should be stopped. An expert consensus defined the role of PCT in surgical patients and IAI. The consensus suggests PCT should be used to guide the duration of antibiotic therapy and to suggest the need for reinterventions in postoperative IAI [28]. However, it has to be mentioned that currently PCT is not recommended to establish the initiation of antibiotic therapy in critically ill patients [25]. C-reactive protein can be also used as a marker to decide an ADE strategy, together with PCT and clinical signs.

## 9. Immune Status

Immuno-compromised patients deserve a dedicated mention when discussing about ADE. These patients could have sepsis or septic shock without any typical warning sign such as leukocytosis or fever. Some examples of these patients are solid organ transplant recipients receiving immunosuppressive medications, haematological solid cancer patients receiving cytoablative chemotherapy and HIV-infected patients. In addition, some authors suggest ageing is associated with a decreased immunity response predisposing these patients to severe bacterial infections [25]. These particular patient are often excluded from the above mentioned studies investigating ADE, especially from randomized trials. Therefore, the findings previously reported on ADE should be applied with caution to these special populations.

## 10. Application of Antibiotic De-Escalation in Emergency General Surgery: Current Evidence in Acute Appendicitis and Acute Cholecystitis

In patients with uncomplicated IAI such as uncomplicated appendicitis and uncomplicated cholecystitis, where the source of infections is treated definitively, postoperative antibiotic therapy is not necessary [29]. Guidelines state accordingly that prolonging antibiotic treatment after pre-operative prophylaxis is unnecessary in uncomplicated acute appendicitis after appendectomy, both in the adult and pediatric population. After appendectomy for complicated acute appendicitis, ADE should be based on clinical and microbiological criteria. However, a period of 3 to 5 days of treatment is generally sufficient and there is no evidence that longer treatments improve postoperative outcomes. A recent RCT compared the outcomes of short (24 h) and extended (>24 h) postoperative antibiotic therapy in complicated AA. The overall rate of complications was 17.9% and 29.3% in the short and extended group, respectively (P = 0.23) [30]. Sawyer et al. enrolled 518 patients with complicated intra-abdominal infection, also including complicated AA, undergoing adequate source control and demonstrated that outcomes after fixed-duration antibiotic therapy (approximately 4 days) were similar to those after a longer course of antibiotics (approximately 8 days) [12].

Similarly, uncomplicated acute calculous cholecystitis patients can be treated without postoperative antibiotics when the focus of infection is controlled by cholecystectomy. In a recently published prospective randomized controlled trial, patients were randomized to either no antibiotics after surgery or continuation with the preoperative antibiotic regimen three times daily for 5 days. The postoperative infection rates were 17% (35/207) in the non-treatment group and 15% (31/207) in the antibiotic group (absolute difference, 1.93%; 95% CI, −8.98 to 5.12%) [31].

## 11. Conclusions

No clear consensus exists on ADE components and various definitions have been used. Moreover, most studies focus on ICU patients and there is a lack of high quality data selectively investigating ADE strategies in EGS. ADE has been reported to be applied in only 40–50% of inpatients with bacterial infection [6]. IAI are known to be a major issue and a call for studies involving ADE strategy in EGS patients is required in the acute care surgery community. Hospitals should establish preferred empiric regimens and de-escalation flow-charts for EGS diseases, taking into account local and national guidelines together with local antimicrobial susceptibilities. At the same time, surgeons should be aware of the strong evidence available supporting ADE strategies and the impact of ADE in improving patients’ clinical outcomes. Consensus definitions are required in order to overcome the low acceptance of recommendations to decrease antibiotic exposure. Indeed, recommendations which aim to reduce antimicrobial exposure have significantly lower odds of acceptance than those which increase exposure among surgical services. Surgeons were significantly less likely to accept recommendations as compared to general medicine providers [4]. The clinical strategy in EGS is often complex and the decision on ADE should be made collaboratively during ward rounds between surgeons, intensivists and infectious disease specialists based on the patient clinical status and microbiological evidence. The implementation of ADE in EGS would be of great importance in improving patients’ clinical outcomes, optimizing antibiotics administration, reducing antibiotic resistance, improving IAI management and, of course, reducing hospital stay and health-care costs.

## Figures and Tables

**Table 1:** Definitions of antibiotic de-escalation.

Author	Definition
Tabah [5]	Narrowing of the spectrum of antimicrobial treatment
Tabah [5]	Decrease in the number of antibiotics used in a treatment regimen or as a shortening in the duration of the therapy
Waele [6]	Stopping combination therapy
Garnacho [7]	Switching antibiotics from intravenous to oral route

**Table 2:** Major studies available investigating ADE in EGS.

Author	Study	Findings
Garnacho-Montero et al. [7]	prospective observational study	Hospital mortality rate was significantly lower in ADE strategy
Turza et al. [11]	Retrospectively	ADE was not associated with an increased mortality as compared to no-ADE strategy
Sawyer et al. [12]	Multicenter, RCT	Similar outcomes were demonstrated after fixed-duration antibiotic therapy (approximately 4 days) as well as after a longer course of antibiotics
Montravers et al. [13]	Multicenter. RCT	No differences in ICU and hospital length of stay, emergence of multi drug resistant bacteria, reoperation rate and mortality between ADE and no-ADE groups
Montravers et al. [14]	Case-control	No differences in Sequential Organ Failure Assessment (SOFA) score changes and mortality between ADE and no-ADE groups; ADE is a feasible option in patients with polymicrobial IAI
Koupetori et al. [15]	Multicenter RCT	No difference between ADE and non-ADE group (2006–2009) and prolonged survival in ADE group (2010–2013)
Tabah et al. [5]	Literature review	A protective effect of ADE
Hayashi et al. [16]	Retrospective analysis	Therapy shorter that 7 days was associated with the same mortality rate and lower recurrence rate than longer therapies
